# Knowledge, Attitudes, and Perceptions of Skin Cancer Clinical Trials in the Hispanic Population

**DOI:** 10.7759/cureus.57480

**Published:** 2024-04-02

**Authors:** Luis F Andrade, Maria J Lalama, Siri Choragudi, Jezabel Maisonet, Juan Ayala, Cesar Figueroa, Liz C Lopez, Lauren Tavarez, Robert S Kirsner, John Strasswimmer

**Affiliations:** 1 Dermatology, Dr. Phillip Frost Department of Dermatology and Cutaneous Surgery, University of Miami Miller School of Medicine, Miami, USA; 2 Medicine, Dr. Phillip Frost Department of Dermatology and Cutaneous Surgery, University of Miami Miller School of Medicine, Miami, USA; 3 Dermatology, Caridad Center, Boynton Beach, USA; 4 Internal Medicine, Cesar Figueroa Clinic, Delray Beach, USA; 5 Dermatology, Florida Atlantic University, Boca Raton, USA; 6 Surgery, Strasswimmer Dermatology, Delray Beach, USA

**Keywords:** hispanic population, health care disparities, ­skin cancer, clinical research, general dermatology

## Abstract

Objective: To determine the factors that might limit Hispanic patients from participating in dermatological clinical trials.

Methods: From January 2022 to July 2022, we administered a 31-item, in-person questionnaire to patients recruited in the waiting area of the Caridad Center, one of the largest free clinics in the United States with a predominately Hispanic population, and a nearby private primary care clinic.

Results: Overall, Hispanic patients agreed significantly more with statements in the domain of attitude and behavioral beliefs compared to non-Hispanic survey respondents. The Hispanic ethnicity was associated with increased odds of agreeing with the following statements: “My community would really benefit from skin cancer clinical trials” (OR=0.52; 95% CI 0.30, 0.92), “My participation in a skin cancer study would be very good” (OR=0.59; 95% CI 0.35, 0.99), and “I like to do good for others” (OR=0.41; 95% CI 0.22, 0.77).

Conclusion: While the United States population is composed of 18.5% Hispanics, they only account for 1% of patients enrolled in clinical trials. This study helps identify potential motivational factors for Hispanic patients to participate in skin cancer clinical trials.

## Introduction

Minority groups in the United States have predominately been underrepresented in clinical trials compared to individuals identifying as non-Hispanic white or Caucasian [1]. Clinical trials provide evidence-based medical options for patients with challenging diseases. They also serve as the backbone of integrating medicine and research by allowing investigators to evaluate interventions in a controlled setting. This is vital in determining treatment safety and efficacy. Many cancers, such as metastatic colorectal cancer, even have recruitment criteria as a third line and beyond [2]. Since clinical trial representation is lacking, the safety and efficacy of therapeutics for underrepresented racial and ethnic patients could be overlooked. For example, a clinical study determined that carbamazepine has an increased incidence of Stevens-Johnson syndrome and toxic epidermal necrosis in individuals of Asian descent. This led to an updated Food and Drug Administration recommendation of genetic screening for patients of Asian ancestry before treatment initiation [3]. Without adequate racial and ethnic representation in clinical trials, key findings, such as adverse effects, could be missed. To ensure high external validity, the patient population in clinical trials should correlate with the patients who will be utilizing these interventions in practice. Additionally, adequate representation in clinical trials could reduce health disparities for minority groups.

Skin cancer is the most common cancer, and melanoma is among the top 10 most prevalent malignancies in the United States [4]. While white patients have the highest prevalence and incidence of melanoma, Hispanic and Black patients have significantly shorter survival along with increased progression to later-stage melanoma [5]. Upon reaching stage III and stage IV disease, referral to a clinical trial is an available treatment recommendation. However, one of the most significant health disparities in clinical trial representation involves the Hispanic population [1]. Although the 2020 United States Census reports Hispanics comprising 18.5% of the United States population, only 1% of all clinical trial participants identified as Hispanic [6]. Moreover, a systematic review of randomized clinical trials (RCTs) showed that approximately 40% of dermatology-related RCTs failed to mention the racial or ethnic demographic of study participants [7].

While dermatology clinical trials have seen an increase in gender representation, racial and ethnic minority representation has yet to reflect the United States population [7]. Barriers to patient enrollment in clinical trials, specifically in the Hispanic and African American populations, include mistrust, lack of comfort with the research process, lack of information, time and resource constraints, and a lack of awareness that clinical trials are potential options [8]. Representation of race and ethnicity is essential in dermatology, as numerous studies have demonstrated substantial healthcare disparities. For example, a retrospective review demonstrated that Black patients had a longer time from diagnosis to definitive surgeries for melanoma compared to non-Hispanic white patients [9]. Moreover, patients with skin of color (including those of Hispanic/Latino, Asian, African, Native American, and Pacific Island origin) present with more frequent and unique dermoscopic features of skin cancers, which could further contribute to health care disparities [10].

To determine what influences participation in skin cancer clinical trials, we conducted a survey to assess potential subjects’ knowledge, attitudes, and perceptions (KAP). This KAP survey compared a potential pool of patients in a suburban private practice setting to patients from a local free clinic. The Caridad Center is one of the largest free clinics in the United States, offering medical, dental, and vision health services, among other opportunities, to a predominately Hispanic population. We have previously carried out a KAP study on concerns about skin cancer, which led to an intervention to improve patient outcomes [11-14]. Our goal is to assess patients' current knowledge and concerns regarding clinical trial participation, better understand barriers at the community health system level that potentially prevent minority access and representation in clinical trials, and serve as a guide for future studies on improving clinical trial diversity.

## Materials and methods

Study design

Following the University of Miami institutional review board approval (approval no. 20210849), from January 2022 through July 2022, we administered a 31-item, in-person questionnaire to patients recruited from the waiting area of the Caridad Center, one of the largest free clinics in the United States with a predominately Hispanic population, and a nearby primary care private clinic. Our 31-item questionnaire was derived and simplified from the Clinical Research Involvement Scales (CRIS), a 41-item instrument created to measure community attitudes toward participation in clinical trials [15]. Frew et al. showed CRIS to be a reliable and valid instrument through a three-phase study that exhibited good internal consistency (α range = 0.730-0.917). The validated questionnaire covers different aspects: normative beliefs, behavioral perspectives, and subjective norms. For each aspect, several statements were developed, and a Likert scale with responses included five categories: (1) strongly agree, (2) agree, (3) neutral, (4) disagree, and (5) strongly disagree. Demographic characteristics collected in the survey include age, sex, race or ethnicity, education level, health insurance coverage, and annual household income.

Sample size determination

The sample size for our study was determined based on the primary objective of assessing the KAP of skin cancer clinical trials among the Hispanic population. We conducted a power analysis to ensure that our study had sufficient power to detect significant differences in KAP between Hispanic and non-Hispanic participants. The analysis indicated that a total sample size of 252 participants would allow us to detect meaningful differences with a power of 80% and a significance level of 5%, considering an effect size derived from preliminary studies in similar settings.

Sampling process

The participants for this study were recruited using a stratified random sampling method to ensure that our sample was representative of the patient population visiting the Caridad Center and a nearby private primary care clinic. The stratification was done based on key demographic variables, including age, sex, and ethnicity, to reflect the diverse characteristics of the population. Potential participants were approached in the waiting area of the clinics, and those who met the inclusion criteria were invited to participate in the study. Eligible respondents were at least 18 years old and English or Spanish speakers. We excluded respondents who were younger than 18 years old or did not speak English or Spanish. All eligible subjects provided verbal consent before the completion of the questionnaires. Participants did not receive compensation for participating in the survey. Our recruitment team consisted of all bilingual staff members to ensure that the language barrier did not hinder participation. Each participant was informed about the purpose of the study, the confidentiality of their responses, and their right to withdraw at any time without any consequences.

To minimize selection bias, the recruitment was conducted on different days of the week and during varying clinic hours. This strategy ensured that our sample included a wide range of patients, including those with appointments on weekends and at different times, thereby enhancing the representativeness of our sample. By incorporating a systematic approach to our sampling process and ensuring a sample size determined through power analysis, we believe that our study findings reliably reflect the KAP of skin cancer clinical trials among the Hispanic population visiting the Caridad Center and the nearby private clinic.

Statistical analysis

Frequencies were conducted to describe the sample and calculate the response percentages for the knowledge and attitude items. All tests were two-tailed, with an alpha criterion of p < 0.05. Statistical analyses were performed twice by two independent investigators to ensure the accuracy of the results. Statistical significance was set at p < 0.05 with a 95% CI for all tests. Surveys with missing responses were included in the analysis without imputing missing values.

## Results

A total of 252 participants completed the questionnaire, and descriptive statistics are summarized in Table 1. Around 78% (n=197) of the participants were from the Caridad Center, and 22% (n=55) were from the private clinic; 62% (n=156) of the participants completed the survey in Spanish; 22.4% (n=56) identified as White, 16% (n=40) as Black, 1.6% (n=4) as Asian, 0.4% (n=1) as Pacific Islander, 1.2% (n=3) as Native American, and 0.4% (n=1) as Other, 58% (n=145) identified as Hispanic/Latino ethnicity. About 80% (n=182) of the participants reported a household income of less than $40,000.

**Table 1:** Descriptive statistics of the KAP survey KAP: Knowledge, attitudes, and perceptions

Variables		n	Percentage
Location	Caridad Center	197	78.2
	Delray Private Clinic	55	21.8
Language	English	96	38.1
	Spanish	156	61.9
Age (years)	18-29	10	4.1
	30-39	45	18.7
	40-49	47	19.5
	50-59	61	25.3
	60	78	32.4
Sex	Male	107	43.3
	Female	140	56.7
Race/ethnicity	White	56	22.4
	Black	40	16.0
	Asian	4	1.6
	Pacific Islander	1	0.4
	Native American	3	1.2
	Hispanic/Latino	145	58.0
	Other	1	0.4
Hispanic ethnicity	Yes	145	58.0
	No	105	42.0
Highest level of education	No Schooling	10	4.1
	Elementary School	25	10.4
	Middle School	25	10.4
	High School	102	42.3
	College	59	24.5
	Graduate Schooling	20	8.3
Household income	Less than $40,000	182	80.2
	$40,001-$60,000	20	8.8
	$60,001-$100,000	17	7.5
	$100,000 and more	8	3.5
Awareness of a clinical trial	Yes	149	60.6
	No	97	39.4
Awareness of skin cancer	Yes	189	76.5
	No	58	23.5
Previously diagnosed with skin cancer?	Yes	7	2.8
	No	240	97.2
Family members/friends diagnosed with skin cancer?	Yes	36	15.0
	No	204	85.0

The associations between Hispanic ethnicity and the KAP survey response scores are summarized in Table 2. Overall, Hispanic patients agreed significantly more with statements in the domain of attitude and behavioral beliefs compared to non-Hispanic survey respondents. Behavioral beliefs allow us to understand a patient’s motivation for joining a clinical trial with respect to the behavior’s outcome [3]. The Hispanic ethnicity was associated with increased odds of agreeing with the following statements: “My community would really benefit from skin cancer clinical trials” (OR=0.52; 95% CI 0.30, 0.92), “My participation in a skin cancer study would be very good” (OR=0.59; 95% CI 0.35, 0.99), and “I like to do good for others” (OR=0.41; 95% CI 0.22, 0.77).

**Table 2:** Associations between Hispanic ethnicity and KAP Survey statements The models were constructed using ordinal logistic regression. Response (1 = strongly agree; 2 = agree; 3 = neutral; 4 = disagree; and 5 = strongly disagree) to the survey statement was defined as the ordinal outcome, and Hispanic ethnicity (Hispanic or non-Hispanic) was defined as the main binary exposure variable. Non-Hispanic ethnicity was the reference category. ^a ^Multivariable ordinal regression was adjusted for age and sex. Bold results are statistically significant. KAP: Knowledge, attitudes, and perceptions

Variables	n	Unadjusted OR (95% CI)	Adjusted OR^a^ (95% CI)
My community would really benefit from skin cancer clinical trials	231	0.45 (0.27, 0.76)	0.52 (0.30, 0.92)
My participation in a skin cancer study would be very good	225	0.61 (0.37, 0.99)	0.59 (0.35, 0.99)
My involvement in this cause will result in more ethical research	222	0.72 (0.45, 1.17)	0.71 (0.42, 1.20)
My involvement in this cause will improve my community's trust in medical research	226	0.60 (0.37, 0.98)	0.63 (0.37, 1.05)
I would participate in sunscreen research trial because it would help to prevent skin cancer	224	0.96 (0.59, 1.57)	1.11 (0.64, 1.90)
My participation in skin cancer research study would be more trouble than what it's worth	220	1.25 (0.78, 1.99)	1.09 (0.66, 1.81)
Even if I wanted to participate in a skin cancer research study, I just don't have the time	223	1.20 (0.75, 1.91)	1.19 (0.72, 1.96)
Participating in a skin cancer trial sounds risky	218	1.28 (0.80, 2.05)	1.24 (0.74, 2.05)
I would participate in a skin cancer research study, but I don't like sharp objects	218	1.00 (0.63, 1.60)	1.01 (0.61, 1.67)
I am concerned that a research trial would cause me to get skin cancer	220	0.92 (0.57, 1.48)	0.90 (0.54, 1.50)
I think my doctor would approve of my involvement in skin cancer research	216	0.97 (0.59, 1.59)	0.86 (0.50, 1.46)
I think my work colleagues would approve of my involvement in skin cancer research	216	1.05 (0.65,1.69)	0.95 (0.57, 1.58)
My immediate family is supportive of my involvement in skin cancer research	220	0.93 (0.58, 1.50)	0.88 (0.53, 1.45)
Most people important to me usually support my interests	218	0.97 (0.59, 1.60)	1.03 (0.60, 1.77)
If my pastor supported skin cancer research trials, I would be inclined to get involved	205	1.17 (0.72, 1.90)	1.30 (0.77, 2.18)
I tend to be concerned about what people think about me, even if I don't know them	218	1.12 (0.70, 1.81)	1.15 (0.69, 1.93)
I generally do what my family expects of me	221	1.22 (0.76, 1.95)	1.20 (0.73, 1.98)
I would not want to do something my friends disapproved of	216	1.60 (0.99, 2.57)	1.59 (0.96, 2.63)
If my superiors told me to do something I disagreed with, I would obey their wishes	216	1.25 (0.77, 2.03)	1.31 (0.78, 2.21)
Sometimes I do what my friends say to do, even though I know they are wrong	212	1.21 (0.74, 1.99)	1.17 (0.69, 1.99)
I like to do good for others	228	0.42 (0.23, 0.74)	0.41 (0.22, 0.77)
Skin cancer is a serious concern in my immediate community	222	0.99 (0.62, 1.60)	0.92 (0.56, 1.54)
Full body skin screening is a benefit of a skin cancer study	224	1.13 (0.68, 1.90)	1.11 (0.63, 1.94)
I would benefit from the medical care associated with a skin cancer study	222	0.95 (0.58, 1.55)	0.83 (0.49, 1.42)
Most people who are important to me think I should participate in skin cancer research effort	217	1.15 (0.72, 1.84)	1.08 (0.65, 1.79)
Most people who are important to me would approve of my involvement in this cause	219	1.34 (0.83, 2.16)	1.15 (0.69, 1.92)
Most people who are important to me would support my interest in this cause	219	1.32 (0.81, 2.14)	1.08 (0.64, 1.83)
Being active with clinical research at the Caridad Center/Figueroa Medical Center would help me to express who I am	213	1.35 (0.84, 2.18)	1.28 (0.77, 2.14)
Hearing that somebody else is involved with clinical research at the Caridad Center/Figueroa Medical Center tells me a lot about that person.	212	1.17 (0.72, 1.89)	1.15 (0.69, 1.92)
Others would view me favorably if I volunteered for a clinical trial study at the Caridad Center/Figueroa Medical Center	211	1.76 (1.08, 2.85)	1.64 (0.98, 2.75)
Being involved with the Caridad Center/Figueroa Medical Center research site helps me feel empowered	211	1.74 (1.08, 2.81)	1.64 (0.98, 2.73)
I feel a sense of belonging through my participation in this effort	211	1.27 (0.79, 2.04)	1.31 (0.66,1.81)
I am advancing the public's health and wellbeing through my support of this cause	210	1.32 (0.80, 2.16)	1.32 (0.78, 2.23)
I feel a sense of purpose in this cause	215	1.32 (0.80, 2.16)	1.27 (0.74, 2.17)

## Discussion

It has been established that racial and ethnic minority groups experience healthcare disparities [1]. The underrepresentation of minority groups in dermatological clinical trials could further contribute to this inequality. Prior studies reporting this have proposed survey studies for “future directions” to elucidate the causes of underrepresentation in clinical trials [1]. Community-based assessments (such as this KAP survey) are useful for quantifying which target areas in a public health intervention would be most effective in addressing disparities in clinical trial representation. Using a questionnaire based on the Theory of Reasoned Action, a concept aimed at explaining the relationship between attitudes and behaviors leading to action, we determined several key points regarding Hispanic attitudes and behaviors leading to the action of participating in dermatological clinical trials.

Future clinical trial recruitment methods could emphasize the beneficial outcomes for the patient’s local community and highlight that every individual’s participation in the trial would be an overall positive experience. The attitude domain is one of the main determinants of how a patient will feel towards clinical trial participation; significantly increased agreement with the statement “I like to do good for others” shows that clinical trial recruitment with an emphasis on outcomes providing altruistic benefit to the good of others can be another element that may increase participation from Hispanic patients [4]. Although Spanish-speaking patients at the free clinic were more than likely to agree with statements of outcome evaluation (“My participation in skin cancer research study would be more trouble than what it's worth”), meaning a higher concern with the logistics of clinical trial involvement versus expected positive outcomes, free clinic participants were still more likely to agree with statements regarding clinical trial participation across multiple domains, including behavioral, normative, and subjective norms, when compared to private practice participants. This signals that while there is concern from Spanish-speaking individuals of low socioeconomic status about the logistics of clinical trial participation, their significantly higher willingness to participate across multiple factors shows that interventions aimed at facilitating accessibility could provide higher participation rates. 

With the percentage of Hispanic people in the U.S. population increasing over time, it is important to focus on key behavioral domains identified in this study for future recruitment methods aimed at increasing Hispanic participation in dermatological clinical trials. As highlighted previously, recruitment efforts demonstrating trial participation as being an altruistic action in nature, a beneficial outcome for the patient’s identified community, and most notably, a positive experience for the patient that is not cumbersome, should be key objectives to incentivize recruitment.

This study has potential limitations. The results are based on an in-person survey and include potential selection and recall biases since only two outpatient clinics were sampled. Future studies could distribute a similar survey throughout multiple clinic spaces and various domains, such as online.

## Conclusions

To our knowledge, this is the first study evaluating Hispanic patients’ attitudes and behaviors toward participating in dermatological clinical trials. We anticipate that, based on this initial KAP analysis, an appropriate intervention can be designed to empower minority patients, specifically Hispanics, to participate in clinical dermatological trials. Programs utilizing Hispanic community health workers to educate local populations about the process and impact of clinical trial participation could be an effective method to reduce the gap in clinical trial representation.
